# Tigecycline resistance among carbapenem-resistant *Klebsiella Pneumoniae*: Clinical characteristics and expression levels of efflux pump genes

**DOI:** 10.1371/journal.pone.0175140

**Published:** 2017-04-07

**Authors:** Sheng-Kang Chiu, Ming-Chin Chan, Li-Yueh Huang, Yi-Tsung Lin, Jung-Chung Lin, Po-Liang Lu, L. Kristopher Siu, Feng-Yee Chang, Kuo-Ming Yeh

**Affiliations:** 1 Division of Infectious Diseases and Tropical Medicine, Department of Internal Medicine, Tri-Service General Hospital, National Defense Medical Center, Taipei, Taiwan, ROC; 2 Graduate Institute of Medical Sciences, National Defense Medical Center, Taipei, Taiwan, ROC; 3 Infection Control Office, Tri-Service General Hospital, National Defense Medical Center, Taipei, Taiwan, ROC; 4 Institute of Infectious Diseases and Vaccinology, National Health Research Institutes, Miaoli, Taiwan, ROC; 5 Section of Infectious Diseases, Department of Medicine, Taipei Veterans General Hospital, National Yan-Ming University, Taipei, Taiwan, ROC; 6 Department of Internal Medicine, Kaohsiung Medical University Hospital, Kaohsiung, Taiwan, ROC; 7 Graduate Institute of Basic Medical Science, China Medical University, Taichung, Taiwan, ROC; Cornell University, UNITED STATES

## Abstract

**Objectives:**

Tigecycline is a treatment option for infections caused by carbapenem-resistant *Klebsiella pneumoniae* (CRKP). Emerging tigecycline resistance in CRKP represents a growing threat. Knowledge of the clinical, microbiological, and molecular characteristics of tigecycline- and carbapenem-resistant *Klebsiella pneumoniae* (TCRKP) is limited.

**Methods:**

Patients infected with TCRKP were identified from a Taiwanese national surveillance study. Clinical data were collected from medical records. We performed susceptibility tests, carbapenemase gene detection, pulsed-field gel electrophoresis (PFGE) and multilocus sequence typing (MLST). Furthermore, we performed quantitative real-time polymerase chain reaction (qRT-PCR) analyses to assess the expression levels of the efflux pump genes *acrB* and *oqxB*.

**Results:**

We identified 16 patients infected with TCRKP, with urinary tract infection (UTI) being the most common type of infection (63%). The all-cause 30-day mortality rate was 44% in patients with TCRKP infection. Patients with a site of infection other than the urinary tract had a significantly higher mortality rate than patients with UTIs (83% vs. 20%, p = 0.035). PFGE and MLST revealed no dominant clone or sequence type. Using qRT-PCR, overexpression of both the *acrB* and *oqxB* genes was identified in seven isolates, and overexpression of the *oqxB* gene was observed in another seven. There was poor correlation between *acrB* or *oqxB* expression and tigecycline MICs (*r* = -0.038 and -0.166, respectively).

**Conclusions:**

The mortality rate in patients infected with TCRKP in this study was 44% and this is an important subset of patients. The absence of a linear relationship between efflux pump genes expression and MICs indicates that tigecycline resistance may be mediated by other factors. Continuous monitoring of tigecycline resistance among CRKP isolates and resistance mechanisms are necessary.

## Introduction

*Klebsiella pneumoniae* is an important pathogen that causes various infections including bacteremia, pneumonia, liver abscesses, and urinary tract infections [1]. The prevalence of carbapenem-resistant *K*. *pneumoniae* (CRKP) has been increasing globally, making antimicrobial treatment difficult and causing higher disease-related mortality rates [2,3]. Tigecycline is one of the few available choices for treating carbapenem-resistant bacterial infections [4]. Once CRKP develops resistance to tigecycline, the treatment options are much more limited.

To date, studies on the clinical characteristics and outcome of tigecycline resistance superimposed to CRKP are very limited [5–7]. The nonsusceptibility rate of tigecycline against CRKP was 9.1% in a surveillance study from Taiwan [8]. The resistance rate of tigecycline among carbapenemase-producing *K*. *pneumoniae* was reported to be 11.2% in China [9] and 14.5% in Korea [10]. In their study, van Duin et al. found that the rate of tigecycline resistance among patients with CRKP was 18% in a multicenter, prospective cohort of hospitalized patients in the USA [7]. The increasing prevalence of tigecycline resistance, after its launch in 2005, is a growing concern [11].

The mechanism of tigecycline resistance is complex and has not yet been fully elucidated [12]. Previous studies have reported that increased expression of efflux pumps such as AcrAB and OqxAB play an essential role in the tigecycline resistance mechanisms of *K*. *pneumoniae* [9,13]. The AcrAB efflux pump is a tripartite complex consisting of the large cytoplasmic membrane protein AcrB, the membrane fusion protein AcrA and a channel, TolC; this pump expels a variety of antibiotics including β-lactams, chloramphenicol, erythromycin fluoroquinolones, fusidic acid and tetracycline [14]. The OqxAB efflux pump is chromosomally located in *K*. *pneumoniae* [15] and requires a functional AcrAB efflux pump, although its natural function remains unknown [11].

In the present study, we collected clinical isolates of carbapenem non-susceptible *K*. *pneumoniae* (CnSKP) between January 2012 and December 2014 in a Taiwanese surveillance study and identified 16 isolates of tigecycline- and carbapenem-resistant *K*. *pneumoniae* (TCRKP). We investigated the clinical characteristics and outcome of patients with TCRKP infection, efflux pumps expression among the TCRKP isolates, and elucidated the possible role of AcrAB and OqxAB efflux pumps in tigecycline-resistant phenotypes.

## Materials and methods

### Hospital settings and bacterial isolates

Consecutive non-replicate clinical isolates of CnSKP were collected from 21 hospitals across Taiwan between January 2012 and December 2014, as part of the Study Group of Carbapenem Resistance in *Klebsiella pneumoniae* in Taiwan.

This study was approved by the Institutional Review Boards of all participating hospitals, including Taipei Veterans General Hospital (VGHIRB No.: 2011-11-001IC), Tri-Service General Hospital (IRB No.: 100-05-205), National Taiwan University Hospital (IRB No.: 201110043RB), Kaohsiung Medical University Chung-Ho Memorial Hospital (KMUH-IRB-20110328), Chang Gung Memorial Hospital (IRB No.: 1003399B), China Medical University Hospital (CMUH IRB No.: DMR100-IRB-214), Chi- Mei Medical Center (IRB No.: 10012–001) and Kaohsiung Armed Forces General Hospital (IRB No.: 100–076). The IRBs waived the need for informed consents (both written and verbal) from source patients of the enrolled bacterial isolates because this was an observational study and involved very minimal risk to the source patients; this waiver does not adversely affect the rights and welfare of the source patients.

Tigecycline resistance was interpreted according to the clinical breakpoints specified by the European Committee on Antimicrobial Susceptibility Testing (EUCAST) (a minimum inhibitory concentration (MIC) > 2.0 mg/L is resistant) [16]. Carbapenem resistance was defined by the Clinical and Laboratory Standards Institute (CLSI) M100-S25 interpretive breakpoints [17]. Clinical isolates of *K*. *pneumoniae* with dual resistance to tigecycline and carbapenem were studied.

The isolates collected from each hospital were sent to the National Health Research Institutes, Miaoli, Taiwan and stored at −70°C in 10% glycerol Luria-Bertani medium before analysis. Species confirmation was performed by standard biochemical methods, on a VITEK® 2 automated system (bioMérieux, Marcy l’Etoile, France). This study was approved by the Review Boards of each hospital.

### Clinical data collection

Medical charts were reviewed to extract patient information, including demographic characteristics, underlying medical conditions, Acute Physiology and Chronic Health Evaluation II (APACHE II) scores at the time of TCRKP identification, and clinical outcomes. Types of infection were defined according to the standardized definitions of the Centers for Disease Control and Prevention/National Healthcare Safety Network [5]. Prior tigecycline treatment was considered significant and was included in our analysis only if: (1) the tigecycline had been administered for at least 3 consecutive days; and (2) the exposure had occurred within 30 days prior to the identification of TCRKP. Appropriate antibiotics treatment was defined as treatment with at least one agent for ≥ 48 h after the isolation of a clinical culture specimen to which the isolate was susceptible in vitro [18].

Clinical outcome was classified as success for patients who had resolution of signs and symptoms that defined the infection and as death for patients who died within 30 days of the onset of TCRKP infection.

### Case-control study

For each TCRKP infection case, one control patient with tigecycline-susceptible CRKP infection was selected from CnSKP patients matched by age, sex and infection type as closely as possible. Clinical information on the TCRKP patients and matched controls were collected from medical records.

### Antimicrobial susceptibility testing

The MICs for tigecycline were determined using the E-test (AB Biodisk, Solna, Sweden) on Mueller-Hinton media. MICs for carbapenems (ertapenem, imipenem, meropenem, and doripenem) and other antimicrobial agents were determined using the broth microdilution method (Sensititre, TREK Diagnostic Systems, Cleveland, OH, USA). Tigecycline and colistin susceptibility were interpreted according to the EUCAST clinical breakpoints [16]. The CLSI M100-S25 interpretive breakpoints were used to interpret the MIC results for all antimicrobial agents studied except tigecycline and colistin [17].

### Detection of genes encoding carbapenemase

Carbapenemase genes (encoding Ambler class A families KPC, NMC, IMI, SME, and GES; Ambler class B families IMP, VIM, NDM, GIM, SPM, and SIM; and Ambler class D family OXA-48-type) were detected using polymerase chain reaction (PCR) analysis [19]. The primers used in this study are listed in S1 Table and the PCR analyses were performed under previously described conditions [8].

The amplicons were sequenced and the entire sequences were compared with those in the National Center for Biotechnology Information (NCBI) database at https://blast.ncbi.nlm.nih.gov/Blast.cgi to determine the molecular type.

### Pulsed-field gel electrophoresis and multilocus sequence typing

Pulsed-field gel electrophoresis (PFGE) was performed for TCRKP isolates. In brief, bacterial chromosomal DNA was digested using *Xba*I (New England Biolabs, Beverly, MA, USA) [20]. Electrophoresis was carried out for 22 h at 14°C with pulse times ranging from 2 to 40 s at 6 V/cm with a Bio-Rad CHEF MAPPER® apparatus (Bio-Rad Laboratories, Richmond, CA, USA). A dendrogram based on the unweighted pair group was generated by previously described methods [21]. Isolates with PFGE profiles exhibiting more than 80% similarity were considered closely related strains.

Multilocus sequence typing (MLST) was performed on TR-CRKP isolates according to the protocol described on the *K*. *pneumoniae* MLST website (https://www.pasteur.fr/fr/recherche). The MLST results were typed using the international *K*. *pneumoniae* MLST database at the Pasteur Institute, Paris, France [22].

### Quantitative real-time PCR

The mRNA expression levels of the efflux pump genes (*acrB* and *oqxB*) were examined using quantitative real-time PCR (qRT-PCR). The experiments were performed using a housekeeping gene (23S) as an internal control, and the fold change of each gene was calculated by dividing the mRNA expression level of the abovementioned genes by that of the 23S gene. cDNA (100 ng) was amplified by PCR with 40 cycles of denaturing (95°C, 15 s), annealing (55°C, 30 s), and extension (72°C, 45 s) using Fast SYBR® Green Master Mix (Applied Biosystems®). Quantitative analysis of the PCR products was carried out by a sequence detector (Model 7500 Fast, Applied Biosystems®) according to the manufacturer’s instructions. The experiments were performed in triplicate. The threshold cycle (Ct) value was defined as the cycle number at which the fluorescence generated within a reaction crossed the threshold value, and the relative Ct value of each target gene was compared to that of the tigecycline-susceptible strain (CG43) [23] as a control (expression = 1) to estimate the fold changes in relative mRNA expression among the samples.

### Statistical analysis

Data analyses were conducted using the statistical package SPSS 13.0 (SPSS Inc., Chicago, Illinois). Univariate analysis was performed by a chi-square test or Fisher’s exact test for categorical variables and Student’s *t* test for continuous variables. A value of p < 0.05 was considered statistically significant. The Pearson product-moment correlation coefficient *(r)* was calculated to measure the linear relationship between two random variables. The plus or minus sign of the correlation coefficient denotes the direction of the relationship, ranging from a strong negative correlation (-1) to a strong positive correlation (+1).

## Results

### Hospital settings and bacterial isolates

Consecutive non-replicate clinical isolates (n = 1093) of CnSKP were collected from 21 hospitals across Taiwan from January 2012 through December 2014. Sixteen isolates of TCRKP were identified from eight participating hospitals, of which ten isolates were from northern Taiwan (Hospital A, D, E and H), three were from central Taiwan (Hospital C and F) and three isolates were from southern Taiwan (Hospital B and G).

### Clinical characteristics of patients with TCRKP infections

The clinical features and outcomes of all patients are summarized in Table 1. The male-to-female ratio was 9:7. The mean age was 64.6 (range, 3–103). Ten patients had type 2 diabetes mellitus, which was the most frequent underlying disease among these TCRKP-infected patients. In general, the patients were critically ill, with a median APACHE II score of 22.3 (range, 7–37). Types of infection included urinary tract infections (9 [56%]), pneumonia (2 [13%]), biliary tract infections (2 [13%]), bacteremia (1 [6%]), and peritonitis (1 [6%]). The source of bacteremia was presumed to be catheter-related in one patient. Seven patients were in the ICU at the time of TCRKP isolation. Four patients had received tigecycline treatment prior to TCRKP isolation. Of these four patients, three received tigecycline treatment for antecedent urinary tract infections and one for an antecedent biliary tract infection.

**Table 1:** Clinical features of patients with tigecycline- and carbapenem-resistant *Klebsiella pneumoniae* (TCRKP) infection and molecular types of the TCRKP isolates.

Patient	Isolate	Hospital	Sex	Age	Underlying Diseases	APACHE Ⅱ Score	Type of Infection	Prior TGC Treatment	Treatment of TCRKP Infection	CarbapenemaseGene	MLST Result	Clinical Outcome	AppropriateAntibioticsTreatment
P1	TR1	A	F	74	CVA, Dementia, DM, HTN	33	UTI	Yes, Combination therapy for UTI	AN, CL, TGC	ND	15	Success	Yes
P2	TR2	A	M	88	COPD, Dementia, DM, HTN	14	UTI	No	CL, DOR, GM, LVX	ND	15	Success	Yes
P3	TR3	B	F	63	CVA, DM, HTN	18	UTI	No	FOF	ND	15	Success	Yes
P4	TR4	C	M	70	Hepatoma	28	BTI	No	ERT, GM, PTZ	ND	307	Death	No
P5	TR5	D	F	51	Lung cancer	8	UTI	No	CAZ	ND	231	Success	No
P6	TR6	E	M	61	CHF, Esophageal cancer, DM, Uremia	24	Pneumonia	No	CL, MEM	ND	1192	Death	Yes
P7	TR7	F	M	85	DM, Prostate cancer	37	UTI	No	TGC	*bla*_IMP-8_	1087	Success	No
P8	TR8	G	M	72	CVA, Cholangiocarcinoma	15	BTI	Yes, Combination therapy for BTI	CL, ERT, FEP	ND	307	Success	Yes
P9	TR9	A	F	34	Liver cirrhosis	14	UTI	Yes, Combination therapy for UTI	CL	ND	1619	Success	No
P10	TR10	E	M	81	CVA, COPD, DM, Prostate cancer	24	UTI	Yes, Monotherapy for UTI	TGC	*bla*_KPC-2_	11	Death	No
P11	TR11	A	F	32	Dilated cardiomyopathy, DM	26	UTI	No	FEP, TGC	ND	15	Death	No
P12	TR12	G	M	3	Acute lymphoblastic leukemia	15	Bacteremia	No	AN, MEM, SXT	ND	711	Death	Yes
P13	TR13	D	M	83	DM, Uremia	33	UTI	No	AN, ETP, PTZ	ND	11	Success	Yes
P14	TR14	H	F	45	Bipolar disorder	22	UTI	No	CL, FEP	ND	1644	Success	Yes
P15	TR15	D	M	88	CAD, DM	37	Pneumonia	No	FEP	ND	1253	Death	Yes
P16	TR16	C	F	103	DM	16	Peritonitis	No	CL, TGC	*bla*_IMP-8_	1565	Death	Yes

AN, amikacin; APACHE II, Acute Physiology and Chronic Health Evaluation II; BTI, biliary tract infection; CAD, coronary artery disease; CAZ, ceftazidime; CHF, congestive heart failure; CL, colistin; COPD, chronic obstructive pulmonary disease; CVA, cerebrovascular accident; DM, diabetes mellitus; DOR, doripenem; ERT, ertapenem; F, female; FEP, cefepime; FOF, fosfomycin; GM, gentamicin; HTN, hypertension; LVX, levofloxacin; M, male; MEM, meropenem; MLST, multilocus sequence typing; ND, not detected; PTZ, piperacillin/tazobactam; SXT, trimethoprim-sulfamethoxazole; TCRKP, tigecycline- and carbapenem-resistant *Klebsiella pneumoniae*; TGC, tigecycline; UTI, urinary tract infection.

Of the 16 patients with TCRKP infection, seven received various colistin-based combination regimens. The 30-day mortality of patients with TCRKP infection was 44% (7/16). Among the patients with urinary tract infections, the mortality rate was relatively low (2/10, [20%]) compared with that of patients with pneumonia, biliary tract infection, bacteremia, or peritonitis. The mortality rate of patients with non-urinary tract infections was 83% (5/6).

To determine the variables associated with 30-day mortality among patients with TCRKP infection, a comparison of the clinical features between survivors and non-survivors 30 days after TCRKP infection is shown in Table 2. Age, sex, underlying diseases, APACHE II score, prior tigecycline treatment, appropriate antibiotics treatment, a MIC for meropenem > 4 mg/L, and a MIC for tigecycline > 4 mg/L were not associated with 30-day mortality. The infection type was reclassified into low-risk infection site (urinary tract infection) and high-risk infection site (all others) [24]. An infection site other than the urinary tract was significantly associated with 30-day mortality when compared with urinary tract infections (p = 0.035).

**Table 2:** Risk factors for 30-day mortality among 16 patients infected with tigecycline- and carbapenem-resistant *Klebsiella pneumoniae*.

Variables	30-Day Survivor (n = 9)	30-Day Non-survivor (n = 7)	p value
Age (years; mean ± SD)	66.1 ± 19.1	62.6 ± 34.6	0.452
Sex			0.286
Female	5	2	
Male	4	5	
Underlying disease			
DM	5	5	0.633
CVA	3	1	0.585
Solid tumor	4	2	0.633
Hematological malignancy	0	1	0.438
Uremia	2	0	0.475
Liver cirrhosis	1	0	1.000
Type of infection			0.035
UTI	8	2	
High-risk infection site^a^ (Non-UTI)	1	5	
APACHE II (mean ± SD)	21.56 ± 10.33	24.29 ± 7.46	0.289
Prior tigecycline treatment	3	1	0.585
Appropriate antibiotics treatment			0.549
Yes	6	4	
No	3	3	
MICs			
Meropenem MIC > 4 mg/L	1	3	0.262
Tigecycline MIC > 4 mg/L	7	3	0.302

APACHE II, Acute Physiology and Chronic Health Evaluation II, BTI, biliary tract infection; CVA, cerebrovascular accident; DM, diabetes mellitus; MIC, minimal inhibitory concentration; SD, standard deviation; UTI, urinary tract infection.

^a^ High-risk infection types: any infection except urinary tract infection.

We attempted to compare urinary and non-urinary infection sources among the 16 patients with TCRKP infection (Table 3). The result also showed a difference in 30-day mortality (20% vs. 83%, p = 0.035). The other variables between the two groups were not significantly different.

**Table 3:** Comparison between urinary and non-urinary source among 16 patients with tigecycline- and carbapenem-resistant *Klebsiella pneumoniae* infection.

Variables	Urinary source (n = 10)	Non-urinary source (n = 6)	p value
Age (years), mean ± SD	63.6 ± 21.7	66.2 ± 34.3	0.856
Male gender, no. (%)	4 (40)	5 (83)	0.145
APACHE II score, mean ± SD	22.9 ± 9.5	22.5 ± 8.9	0.935
Prior tigecycline treatment, no. (%)	3 (30)	1 (17)	1.000
Appropriate antibiotics treatment, no. (%)	5 (50)	5 (83)	0.307
30-day mortality, no. (%)	2 (20)	5 (83)	0.035

APACHE II, Acute Physiology and Chronic Health Evaluation II; SD, standard deviation.

### Case-control study

A comparison of variables between patients in case and control group is shown in Table 4. The age, sex, infection type and APACHES II score between the two groups were not significantly different. The mortality for tigecycline-susceptible carbapenem-resistant *K*. *pneumoniae* infection was 31%. Compared with the case group, there was no significant difference in 30-day mortality between the two groups (p = 0.716). The 30-day mortality attributable to tigecycline resistance was 13%.

**Table 4:** Case-control analysis for 30-day mortality between patients with tigecycline-resistant and tigecycline-susceptible carbapenem-resistant *Klebsiella pneumoniae* infection.

Variables	Tigecycline-resistant(n = 16)	Tigecycline-susceptible(n = 16)	p value
Age (years), mean ± SD	64.6 ± 26.0	66.3 ± 20.1	0.833
Male gender, no. (%)	9 (56)	9 (56)	1.000
Type of infection, no. (%)			1.000
UTI	10 (63)	10 (63)	
Non-UTI	6 (38)	6 (38)	
APACHE II score, mean ± SD	22.8 ± 9.0	22.1 ± 11.2	0.863
Appropriate antibiotics treatment, no. (%)	10 (63)	9 (56)	1.000
30-day mortality, no. (%)	7 (44)	5 (31)	0.716

APACHE II, Acute Physiology and Chronic Health Evaluation II; SD, standard deviation; UTI, urinary tract infection.

### Antimicrobial susceptibility and carbapenemase-encoding genes

The MIC values of various antimicrobial agents against the TCRKP isolates are listed in S2 Table. The MICs of tigecycline against these isolates ranged from 4–32 mg/L. All isolates (16/16) were resistant to tigecycline, imipenem and ceftazidime. The susceptibility rates to ertapenem and meropenem were 13% (2/16). The susceptibility rates to doripenem and aztreonam were 25% (4/16). The susceptibility rate to cefepime was 19% (3/16). The susceptibility rates to gentamicin and amikacin were 38% (6/16) and 100% (16/16), respectively. The susceptibility rates to ciprofloxacin and levofloxacin were 13% (2/16). The susceptibility rate to trimethoprim-sulfamethoxazole was 13% (2/16). The susceptibility rate to colistin was 100% (16/16).

Carbapenemase genes were detected in 19% (3/16) of isolates collected, with *bla*_KPC-2_ found in one isolate and *bla*_IMP-8_ in two isolates.

### PFGE analysis and MLST

**Fig 1:**
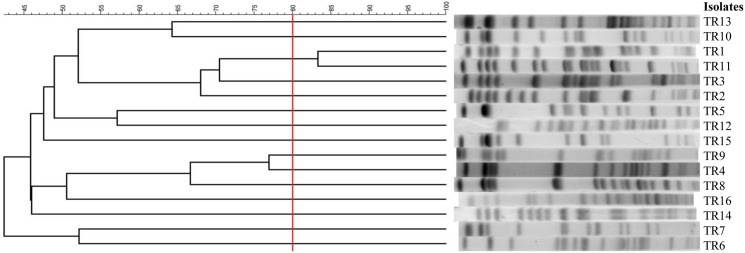
Pulsed-field gel electrophoresis profiles and dendrogram of tigecycline- and carbapenem-resistant *Klebsiella pneumoniae* isolates. Fifteen pulsotypes are shown, using 80% similarity as the cut-off, demonstrating substantial diversity.

The PFGE patterns of the 16 isolates were clustered into 15 pulsotypes at the similarity level of >80%, with more than three bands different from each other, as shown in Fig 1. A wide diversity of PFGE patterns was found, with the exception of two isolates (TR1 and TR11) with the same PFGE band pattern.

Of the 16 isolates, 11 different sequence types were identified, 25% were sequence type ST15 (4/16), 13% were ST11 (2/16), and 13% were ST307 (2/16). Other ST types (ST231, 711, 1087, 1192, 1253, 1565, 1619, and 1644) related to only one isolate each (Table 1). ST1619 and ST1644 were newly-typed MLST types in this study, and the isolate record was sent to the *K*. *pneumoniae* MLST database.

### Efflux pump expression in TCRKP isolates

**Fig 2:**
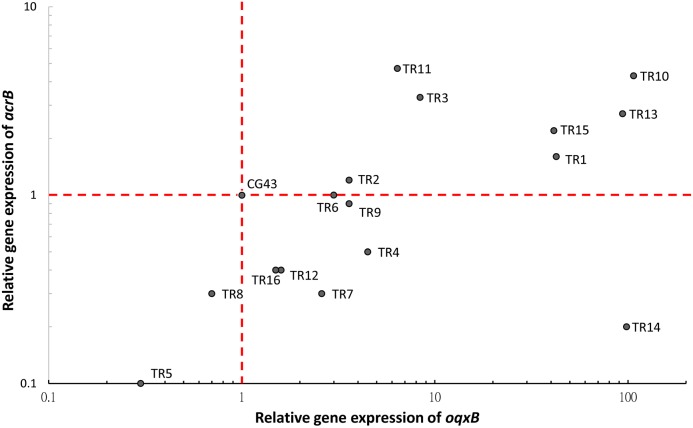
Relative gene expression of *acrB* and *oqx*B in tigecycline- and carbapenem-resistant *Klebsiella pneumoniae* isolates. Overexpression of both *acrB* (1.6- to 4.7-fold) and *oqxB* (3.6- to 107.1-fold) was observed in 7 isolates. Overexpression of *oqxB* (1.5- to 98.5-fold) but not of *acrB* was observed in 7 isolates. No upregulation of *acrB* and *oqxB* in was observed in 2 isolates (TR5 and TR8). Relative gene expression compared with CG43 (expression = 1).

The expression of tigecycline resistance-related efflux pump genes such as *acrB*, and *oqxB* were altered in these isolates compared with the reference strain, CG43 (Fig 2). The expression of both *acrB* and *oqxB* increased in seven isolates (TR1, 2, 3, 10, 11, 13 and 15), while in another seven isolates (TR4, 6, 7, 9, 12, 14 and 16) only the expression of *oqxB* increased. These data indicated that tigecycline resistance in 14 isolates may have been caused by increased expression of *acrB* and/or *oqxB*. However, overexpression of *acrB* and/or *oqxB* could not be detected in two isolates (TR5 and TR8). Taken together, these data indicated that tigecycline resistance mechanisms were not limited to the overexpression of *acrB* and *oqxB*.

**Fig 3:**
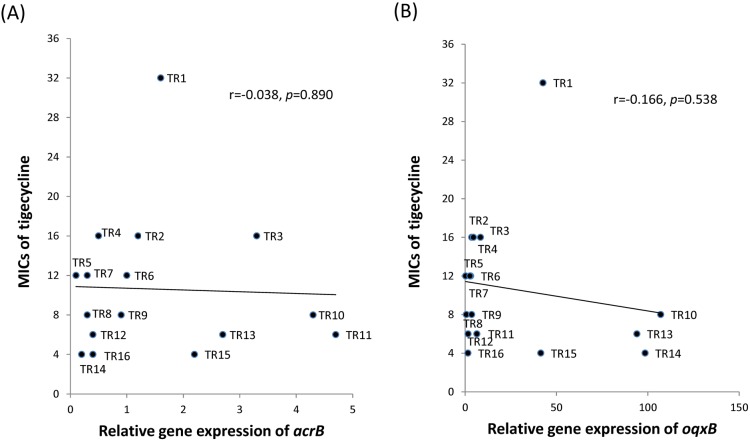
Correlation of gene expression with the minimum inhibitory concentration (MIC) of tigecycline in tigecycline- and carbapenem-resistant *Klebsiella pneumoniae* isolates. A scatter plot displays the poor correlation between tigecycline MIC values and the gene expression of *acrB* (A) and *oqxB* (B).

To evaluate the relationship between the MIC of tigecycline and the relative gene expression levels of *acrB* and *oqxB*, the Pearson product-moment correlation coefficient *(r)* was calculated. However, there was no positive correlation between tigecycline MIC and the gene expression of *acrB* (*r* = -0.038, *p* = 0.89) or *oqxB* (*r* = -0.166, *p* = 0.538) in theses TCRKP isolates (Fig 3).

## Discussion

To the best of our knowledge, the current case series is the first to analyze TCRKP infection with a focus on clinical characteristics and outcome in Taiwan. This study highlight the 30-day mortality rate among patients with TCRKP infection was 44%. Patients with an infection site other than the urinary tract were significantly associated with 30-day mortality when compared with urinary tract infection. The findings from this study are representative of the clinical and molecular characteristics of TCRKP in Taiwan. The emergence of tigecycline resistance in CRKP in Taiwan may be underestimated, as hospitals do not routinely perform tigecycline susceptibility testing against *K*. *pneumoniae*.

Since four patients in this study had previously been exposed to tigecycline before the isolation of TCRKP, this suggests that tigecycline therapy may be a risk factor for developing tigecycline resistance. Long-term tigecycline monotherapy may carry a higher risk for developing tigecycline resistance [11]. A nested case-control study had found that receipt of tigecycline was the independent predictor for subsequent isolation of a tigecycline-resistant *K*. *pneumoniae* isolates [25]. In their study, van Duin et al. described that the use of tigecycline in patients with CRKP bacteriuria was significantly associated with the subsequent development of tigecycline resistance [6]. In contrast, since 75% of the patients in this study were not exposed to tigecycline before TCRKP identification, this suggests that tigecycline resistance may occur without exposure to tigecycline. A previous report suggested that an elevated MIC of tigecycline might be attributed indirectly to the use of other antibiotics that are also effluxed through the AcrAB–TolC pump [26].

This study showed that the overall 30-day mortality rate of patients infected with TCRKP was 44%. Patients with TCRKP bacteremia, pneumonia and peritonitis had very high mortality rates. However, patients with urinary tract infections had a lower mortality rate. Multidrug-resistant *K*. *pneumoniae* infections have been reported to have high attributable mortality rates of up to 50% [27]. The source of infection, severity of underlying conditions, severity of sepsis and appropriateness of the definitive antibiotic therapy were identified as predictors of mortality in a previous study of infections caused by carbapenemase-producing Enterobacteriaceae [24]. Our study also demonstrated that the infection site is important for mortality prediction. However, other predictors could not be identified in our study, which may be due to the small sample size. A large-scale clinical study evaluating the risk factors of mortality and attributable mortality of TCRKP infections is needed. A recent study disclosed that a positive culture of CnSKP was associated with high in-hospital mortality, regardless of colonization or infection [28]. Patients with TCRKP colonization would also likely exhibit high mortality rates. It is difficult to distinguish between colonization and infection when TCRKP isolates are obtained from non-sterile sites in clinical practice. Patients with a positive TCRKP culture, regardless of colonization or infection, may be an emerging challenge for physicians.

According to the susceptibility results of our study, colistin and amikacin have good in vitro activity against TCRKP, with susceptibility rates of 100%. Other classes of effective antibiotics should be considered according to individual susceptibility results. Combination treatment with two or more drugs with in vitro activity against *K*. *pneumoniae* carbapenemase (KPC) -producing *K*. *pneumoniae* isolates has been suggested to improve survival and may be more effective than monotherapy [27]. Combination treatment for patients infected with TCRKP in a high-risk site seems reasonable considering the high mortality rate under this circumstance. A therapeutic strategy leading to a better outcome for patients with TCRKP infection requires further study.

Of the 16 isolates in our study, only two isolates (TR1 and TR11) exhibited the same PFGE band pattern. Though they originated from the same hospital (hospital A), these two infected patients did not have overlapping periods of hospitalization. Intra-hospital spread of TCRKP in hospital A is improbable. Furthermore, as the PFGE patterns in the strains from the other 14 patients were all different, there is little evidence of inter-hospital spread. In summary, we hypothesize that tigecycline resistance among CRKP predominately arises from sporadic cases, and no horizontal spread occurred in this study period. A study conducted in Shanghai, China identified 24 distinct pulsotypes among 26 tigecycline-nonsusceptible *K*. *pneumoniae* clinical isolates [5], which was similar to the results of our study. To prevent the spread of TCRKP, infection control strategies such as standard and contact precautions should be enhanced. Prudent use of tigecycline and other antibiotics that might cause efflux-mediated resistance is also important to prevent the development of tigecycline resistance in *K*. *pneumoniae*.

Our MLST results revealed that the genetic background of TCRKP is heterogeneous in Taiwan. Among the 16 isolates, the strain with the highest tigecycline MIC belonged to ST15, and the predominant ST11 type was not found. KPC-producing *K*. *pneumoniae* ST11 has been reported as the predominant clone of carbapenem-resistant *K*. *pneumoniae* in China [29], Singapore [30], and Taiwan [8]. Our findings suggest that the development of tigecycline resistance in CRKP is unrelated to the local, epidemic ST11 that produces KPC.

The expression levels of tigecycline resistance-related efflux pump genes were determined, and we found that tigecycline resistance could be associated with overexpression of the *acrB* and/or *oqxB* genes in most isolates. A correlation analysis between the expression of efflux pump genes (*acrB* and *oqxB*) and tigecycline MIC indicated a poor correlation. Our findings have suggested that an alternative pathway other than AcrAB or OqxAB regulating tigecycline resistance in these strains. A literature review showed that reported mechanisms in tigecycline resistance of *K*. *pneumoniae* include overexpression of *ramA* and *acrAB* [9, 31, 32], deletions, insertions and point mutations in *ramR* [33, 34], overexpression of *rarA* and *oqxB* [9, 32, 35], a point mutation in the repressor *oqxR* and overexpression of the OqxAB efflux pump [13], IS*5* element integration in a putative efflux pump, KpgABC [23], overexpression of *marA* and *acrAB*, and structural alteration of the ribosomal protein S10 [34]. The detailed role of each mechanism in conferring tigecycline resistance requires further investigation.

There are some limitations of our study. Our data were obtained from multiple centers in Taiwan; hence, the findings may reflect the status of Taiwanese patients, but cannot be generalized to patients in other countries. The patients with TCRKP infections had higher 30-day mortality than those with tigecycline-susceptible CRKP infections. However, there was no statistical significance. Our cases and controls numbers were relatively small to achieve enough statistical power. We need continue to collect data and sample from TCRKP and CnSKP patients for use in future studies. Thus, our investigation of the mechanisms of tigecycline resistance is limited to *acrB* and *oqxB*, which account for few elements of tigecycline resistance, but not all mechanisms of resistance. Transcriptional regulators e.g., RamA, MarA, SoxS and RarA, directly regulate genes linked to efflux pump-mediated resistance to tigecycline, and modulate genes linked to virulence [35–38]. Further study is mandatory to assess these regulators to determine their possible regulatory roles in the microbial response to antimicrobial challenges.

TCRKP that exhibits multidrug resistance reduces therapeutic options and may lead to untreatable infections. Therefore, efforts are urgently needed to improve the knowledge of the epidemiological status of tigecycline resistance accompanied by carbapenem resistance in order to tackle its spread.

## Supporting information

S1 Table Oligonucleotide primer sequences used in this study. (DOC)

S2 Table Minimum inhibitory concentrations of tigecycline- and carbapenem-resistant *Klebsiella pneumoniae* isolates. (DOC)
