# Supplementary material for: Tigecycline resistance among carbapenem-resistant Klebsiella Pneumoniae: Clinical characteristics and expression levels of efflux pump genes
Source: PLoS One. 2017 Apr 7;12(4):e0175140. doi: 10.1371/journal.pone.0175140 (PMC5384758; doi:10.1371/journal.pone.0175140)
Supplement: S1 Table — (DOC) [file pone.0175140.s001.doc]

**S1 Table.** Oligonucleotide primer sequences used in this study

| PCR | Primer name | Sequence (5′-3′) | Reference | |
| --- | --- | --- | --- | --- |
| **Genes encoding class A carbapenemases** | | | | |
| KPC | KPC forward | ATGTCACTGTATCGCCGTCT | 19 | |
|  | KPC reverse | TTTTCAGAGCCTTACTGCCC |  | |
| NMC | NMC1 | GCATTGATATACCTTTAGCAGAGA | 19 | |
|  | NMC4 | CGGTGATAAAATCACACTGAGCATA |  | |
| IMI | IMI-A | ATAGCCATCCTTGTTTAGCTC | 19 | |
|  | IMI-B | TCTGCGATTACTTTATCCTC |  | |
| SME | IRS-5 | AGATAGTAAATTTTATAG | 19 | |
|  | IRS-6 | CTCTAACGCTAATAG |  | |
| GES | GES-C | GTTTTGCAATGTGCTCAACG | 19 | |
|  | GES-D | TGCCATAGCAATAGGCGTAG |  | |
| **Genes encoding class B metalloenzymes** | | | | |
| IMP-1 | IMP-1-F | TGAGCAAGTTATCTGTATTC | | 19 |
|  | IMP-1-R | TTAGTTGCTTGGTTTTGATG | |  |
| IMP-2 | IMP-2-F | GGCAGTCGCCCTAAAACAAA | | 19 |
|  | IMP-2-R | TAGTTACTTGGCTGTGATGG | |  |
| VIM-1 | VIM-1-F | TTATGGAGCAGCAACCGATGT | | 19 |
|  | VIM-1-R | CAAAAGTCCCGCTCCAACGA | |  |
| VIM-2 | VIM-2-F | AAAGTTATGCCGCACTCACC | | 19 |
|  | VIM-2-R | TGCAACTTCATGTTATGCCG | |  |
| SPM-1 | SPM-1F | CCTACAATCTAACGGCGACC | | 19 |
|  | SPM-1R | TCGCCGTGTCCAGGTATAAC | |  |
| GIM-1 | GIM-1F | AGAACCTTGACCGAACGCAG | | 19 |
|  | GIM-1R | ACTCATGACTCCTCACGAGG | |  |
| SIM-1 | SIM1-F | TACAAGGGATTCGGCATCG | | 19 |
|  | SIM1-R | TAATGGCCTGTTCCCATGTG | |  |
| NDM | NDM-F | TCTCGACAATGCCGGGTTT | | In this study |
|  | NDM-R | GAGATTGCCGAGCGACTT | |  |
| **Genes encoding class D oxacillinases** | | | | |
| OXA-48-type | OXA-48-F | TTGGTGGCATCGATTATCGG | | 19 |
|  | OXA-48-R | GAGCACTTCTTTTGTGATGGC | |  |
| **Real-time PCR** | | | | |
| *acrB* | *acrB-*F | CGCCAGGCAAGAGTACCG | | In this study |
|  | *acrB-*R | CGCCAGACACAGGAAGACGA | |  |
| *oqxB* | *oqxB-F* | GAGGTCTCCGGGCCGAT | | In this study |
|  | *oqxB-R* | GGCACGAACACCGCACA | |  |
| *23S* | *23S*-F | GGTAGGGGAGCGTTCTGTAA | | In this study |
|  | *23S*-R | TCAGCATTCGCACTTCTGAT | |  |
