# Supplementary material for: Tigecycline resistance among carbapenem-resistant Klebsiella Pneumoniae: Clinical characteristics and expression levels of efflux pump genes
Source: PLoS One. 2017 Apr 7;12(4):e0175140. doi: 10.1371/journal.pone.0175140 (PMC5384758; doi:10.1371/journal.pone.0175140)
Supplement: S2 Table — (DOC) [file pone.0175140.s002.doc]

**S2 Table. Minimum inhibitory concentrations of tigecycline- and carbapenem-resistant *Klebsiella pneumoniae* isolates**

| Isolate | MIC, mg/L | | | | | | | | | | | | | |
| --- | --- | --- | --- | --- | --- | --- | --- | --- | --- | --- | --- | --- | --- | --- |
|  | TGC | ERT | IPM | MEM | DOR | ATM | CAZ | FEP | GM | AN | CIP | LVX | SXT | CL |
| TR1 | 32 | >4 | 4 | **1** | **1** | >16 | >16 | >16 | **<2** | **<8** | >2 | >4 | >2 | **0.19** |
| TR2 | 16 | >4 | >4 | 2 | 2 | >16 | >16 | >16 | **<2** | **<8** | >2 | >4 | >2 | **0.25** |
| TR3 | 16 | >4 | >4 | 2 | >2 | >16 | >16 | >16 | >8 | **<8** | >2 | >4 | >2 | **0.19** |
| TR4 | 16 | >4 | 2 | >4 | >2 | >16 | >16 | >16 | >8 | **<8** | >2 | >4 | >2 | **0.19** |
| TR5 | 12 | >4 | 4 | **1** | >2 | **4** | 16 | **<2** | >8 | **<8** | >2 | >4 | >2 | **0.19** |
| TR6 | 12 | >4 | >4 | 4 | >2 | >16 | >16 | >16 | >8 | **<8** | >2 | >4 | >2 | **0.19** |
| TR7 | 12 | >4 | 2 | >4 | >2 | **<2** | >16 | >16 | **2** | **8** | >2 | 4 | >2 | **0.25** |
| TR8 | 8 | >4 | >4 | 4 | 4 | >16 | >16 | >16 | >8 | **8** | >2 | >4 | >2 | **0.19** |
| TR9 | 8 | >4 | 2 | 2 | 2 | **4** | 16 | >16 | **<2** | **<8** | **0.25** | **1** | >2 | **0.19** |
| TR10 | 8 | >4 | >4 | >4 | >2 | >16 | >16 | >16 | **<2** | **<8** | >2 | >4 | >2 | **0.19** |
| TR11 | 6 | >4 | >4 | 2 | 2 | **2** | >16 | 8 | **2** | **16** | >2 | >4 | **1** | **0.25** |
| TR12 | 6 | >4 | >4 | >4 | >2 | >16 | >16 | >16 | >8 | **16** | >2 | >4 | >2 | **0.094** |
| TR13 | 6 | **0.5** | 4 | **<0.5** | **0.25** | >16 | >16 | >16 | >8 | **<8** | >2 | >4 | **2** | **0.19** |
| TR14 | 4 | >4 | >4 | 2 | **1** | >16 | 8 | **<2** | >8 | **<8** | >2 | >4 | >2 | **0.25** |
| TR15 | 4 | **0.5** | 4 | **<0.5** | **1** | 16 | 16 | **<2** | >8 | **<8** | **1** | **1** | >2 | **0.125** |
| TR16 | 4 | 2 | 4 | 4 | >2 | >16 | >16 | >16 | >8 | **<8** | 2 | >4 | >2 | **0.094** |

AN, amikacin; ATM, aztreonam; CAZ, ceftazidime; CIP, ciprofloxacin; CL, colistin; DOR, doripenem; ERT, ertapenem; FEP, cefepime; GM, gentamicin; IPM, imipenem; LVX, levofloxacin; MIC, minimum inhibitory concentration; MEM, meropenem; SXT, trimethoprim-sulfamethoxazole; TGC, tigecycline. MICs in susceptible ranges according to the Clinical and Laboratory Standards Institute breakpoints (expect tigecycline and colistin) are shown in bold. Tigecycline and colistin susceptibility was interpreted according to the European Committee on Antimicrobial Susceptibility Testing’s clinical breakpoints. MICs of trimethoprim-sulfamethoxazole were presented according to the concentration of trimethoprim.
